# Case report: mRNA-1273 COVID-19 vaccine-associated myopericarditis: Successful treatment and re-exposure with colchicine

**DOI:** 10.3389/fcvm.2023.1135848

**Published:** 2023-04-17

**Authors:** Luca Valore, Till Junker, Eva Heilmann, Christine S. Zuern, Matthias Streif, Beatrice Drexler, Christian Arranto, Jörg P. Halter, Christoph T. Berger

**Affiliations:** ^1^ Hematology, University Hospital Basel, Basel, Switzerland; ^2^ Hematology, Cantonal Hospital Aarau, Aarau, Switzerland; ^3^ Cardiology, University Hospital Basel, Basel, Switzerland; ^4^ Cardiovascular Research Institute Basel, University of Basel, Basel, Switzerland; ^5^ Radiology, University Hospital Basel, Basel, Switzerland; ^6^ Translational Immunology, University Basel, Basel, Switzerland; ^7^ University Center for Immunology, University Hospital Basel, Basel, Switzerland

**Keywords:** mRNA vaccine, myocarditis, colchicine, allogeneic stem cells transplantation, COVID-19, mRNA-1273

## Abstract

**Introduction:**

Vaccine-induced myocarditis is a rare complication of messenger RNA (mRNA) COVID-19 vaccines.

**Case presentation:**

We report a case of acute myopericarditis in a recipient of allogeneic hematopoietic cells following the first dose of the mRNA-1273 vaccine and the successful administration of a second and third dose while on prophylactic treatment with colchicine to successfully complete the vaccination.

**Conclusion:**

Treatment and prevention of mRNA-vaccine-induced myopericarditis represent a clinical challenge. The use of colchicine is feasible and safe to potentially reduce the risk of this rare but severe complication and allows re-exposure to an mRNA vaccine.

## Introduction

Vaccine-induced myocarditis is a rare complication of messenger RNA (mRNA) vaccines against SARS-CoV-2. There are no reliable data about this adverse event in patients who received allogeneic hematopoietic cell transplantation (HCT). We report a case about a severe clinical course of mRNA-vaccine-induced myopericarditis, its challenging diagnostic process and plausible pathological background, the therapeutical use of colchicine in the acute phase of the disease, and also as a successful prophylaxis by re-exposure to mRNA vaccine against SARS-CoV-2.

## Case presentation

A 70-year-old patient presented to the emergency department complaining of severe back pain radiating to the chest and dyspnea occurring within 12 h following the first dose of the mRNA-1273 vaccine (Spikevax, Moderna). Twenty months earlier, the patient underwent allogeneic hematopoietic cell transplantation for primary myelofibrosis. At the time of presentation, he was in complete remission without signs of active graft versus host disease (GvHD) or infectious disease. Current medication included bisoprolol, ursodeoxycholic acid, budesonide, esomeprazole, insulin, valaciclovir, co-trimoxazole, and gabapentin.

**Figure 1:**
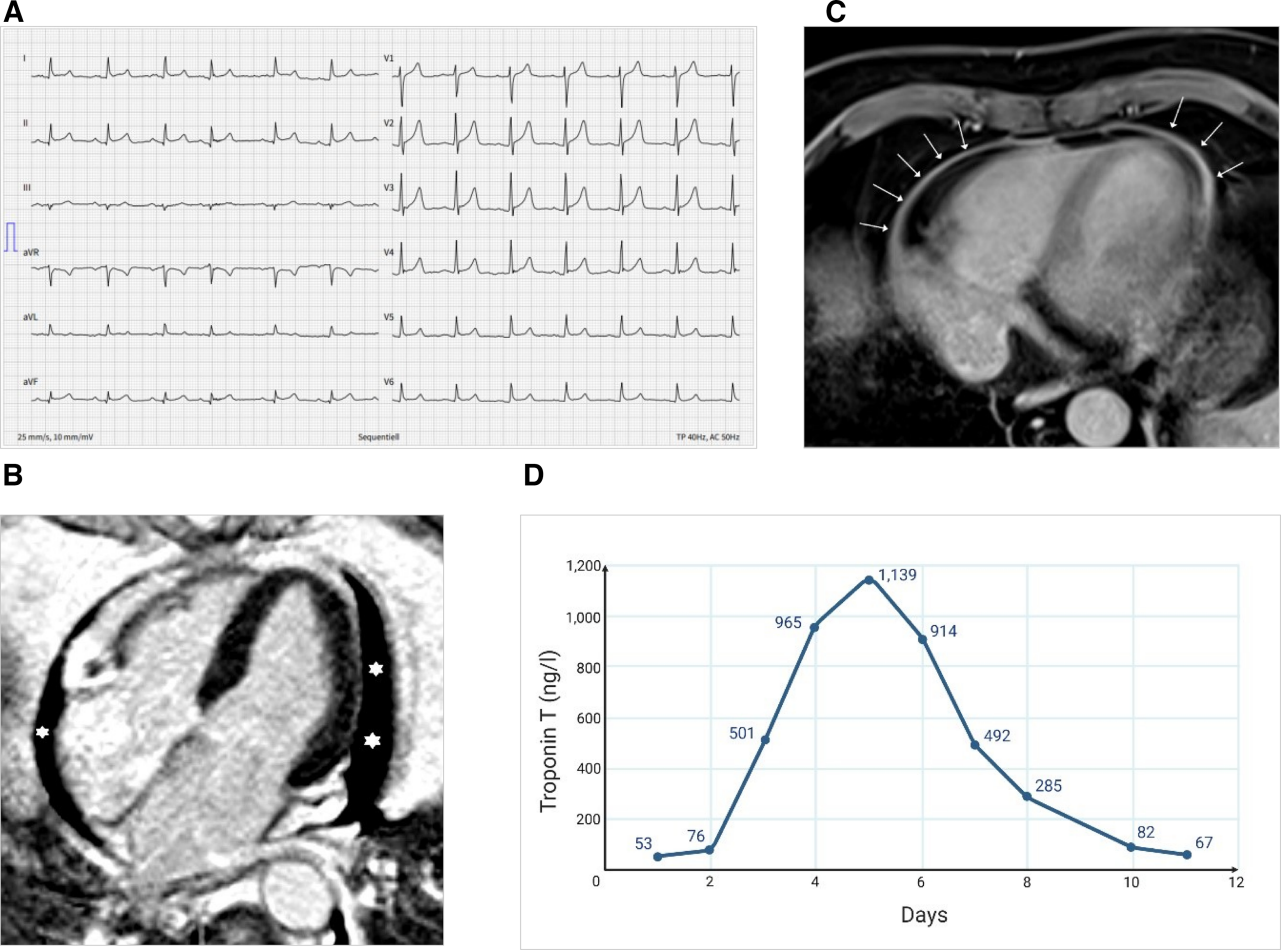
Electrocardiographic and imaging findings at admission and course of high-sensitive troponin T. (**A**) ECG showing diffuse concave ST-elevation (II, aVF, V3-6) and PQ-depression (II, aVF). (**B**) MRI showing pericardial effusion (asterisks) in the four-chamber view (late enhancement PSIR). (**C**) MRI showing pericardial enhancement (arrows) (axial T1 DIXON Water only, which is a fat-saturated, non-ECG-triggered). (**D**) Longitudinal evolution of the high-sensitive troponin T levels during the hospitalization (MRI done on day 3; i.e., troponin 501 ng/L).

On admission, he was hemodynamically stable (blood pressure 136/79 mmHg, heart rate 79 bpm), afebrile, and adequately oxygenated (oxygen saturation 99% in ambient air). Besides arterial hypertension, no other cardiovascular risk factors were present. An electrocardiogram (ECG) showed concave ST-elevation and PQ-segment depression in several ECG leads and not related to a single coronary artery (Figure 1A). Initial laboratory tests indicated a mildly elevated high sensitivity Troponin T of 53 ng/L (reference <14 ng/L), and an N-terminal prohormone of brain natriuretic peptide (NTproBNP) of 655 ng/L (reference <124 ng/L). The remaining laboratory values are summarized in Table 1.

**Table 1:** Laboratory values at admission.

Laboratory investigation	Results	Reference range
Hemoglobin (g/L)	129	120–160
Leucocytes (G/L)	6.3	3.5–10.0
Neutrophils (G/L)	4.7	1.3–6.7
Lymphocytes (G/L)	0.8	0.9–3.3
Platelets (G/L)	153	150–450
C-reactive protein (mg/L)	18.2	<10.0
Creatine kinase (U/L)	29	38–157
Creatine kinase myocardial band (μg/L)	2.5	<5.0
Troponin T high sensitivity (ng/L)	53	<14
N-terminal prohormone of brain natriuretic peptide (NTproBNP) (ng/L)	655	<125
Alanine transaminase (U/L)	93	8–41
Lactate dehydrogenase (U/L)	223	135–214

Aortic dissection, pulmonary embolism, and type 1 myocardial infarction were ruled out by computed tomography scan and coronary angiography, respectively. A single-vessel coronary artery disease with a non-critical right coronary artery stenosis was identified that was not assumed to be responsible for the acute symptoms. Bedside echocardiography showed a hemodynamically insignificant pericardial effusion with preserved left systolic and right heart function bedside echocardiography. A cardiac magnetic resonance imaging (MRI) performed 2 days after symptom onset confirmed pericardial effusion and showed a circumferential pericardial contrast enhancement compatible with pericarditis; the myocardium showed no late gadolinium enhancement and no edema (Figures 1B,C). However, based on the dynamical rise of troponin levels (peak 1,139 ng/L on day 5), a substantial myocardial injury was present. Combined with the ECG changes and cardiac imaging findings and after ruling out alternative causes, acute mRNA vaccine-related probable myopericarditis was diagnosed. No infections or other medical problems in the weeks prior to the presentation could be identified in the patient's history. A nasopharyngeal swab for respiratory viruses, an autoimmune serology panel, and serological tests for enterovirus and adenovirus antibodies were repeatedly negative. A nasal swab and a negative anti-SARS-CoV-2-Nucleoprotein-IgG/M excluded an acute or previous infection with SARS-CoV-2.

The patient was admitted to the hospital for telemetric monitoring and treatment with a non-steroidal anti-inflammatory drug (NSAID) as well as colchicine 0.5 mg every 12 h without systemic steroids was started.

After 13 days, he was discharged (Figure 1D). After 2 months, colchicine was stopped, followed by symptom recurrence after 2 weeks. Troponin was negative then, but C-reactive protein was 16 mg/L (reference <10 mg/L). Echocardiography and a computed tomography scan indicated increased pericardial effusion; thus, a second episode of isolated pericarditis was diagnosed. Therapy with NSAIDs and colchicine was reintroduced and could finally be tapered after 6 weeks without relapse.

**Figure 2:**
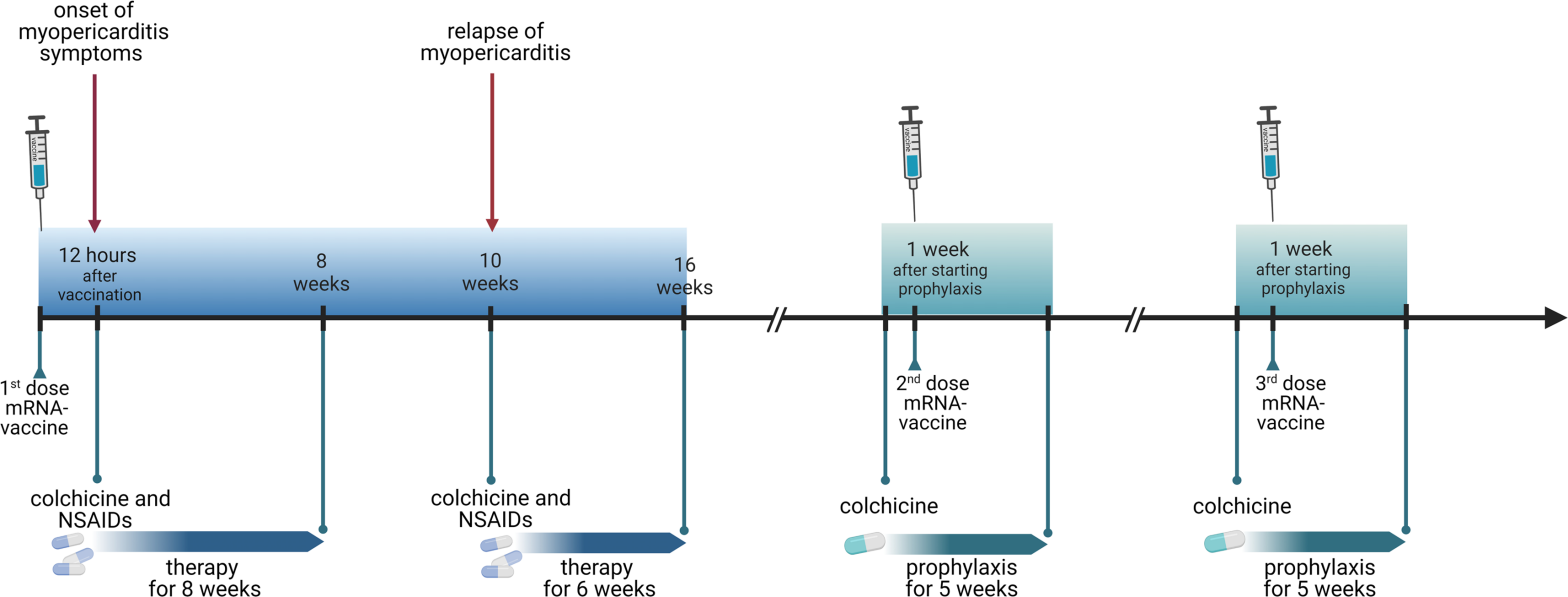
Timeline of clinical course (figure created with biorender.com).

Due to the non-availability of alternative (non-mRNA) vaccines in Switzerland at that time and due to the high individual risk for severe COVID in this patient, we discussed a re-exposure with a second dose of an mRNA vaccine to complete the immunization. In order to prevent the re-occurrence of myopericarditis, we initiated a prophylactic therapy with colchicine 0.5 mg every 12 h, starting a week before the second dose. The rationale for using an inflammasome-inhibiting drug as prophylaxis was based on the data suggesting inflammasome-induced inflammation in the pathogenesis of mRNA vaccine-associated myocarditis (1), and data indicating a reduced risk of (inflammasome mediated) mRNA vaccine induced gout flares on colchicine treatment (2). Because the symptoms developed exceptionally rapidly after the first dose, we opted to monitor the patient in the hospital for 72 h. He received the second dose of mRNA-1273 in September 2021, 5 months after the first dose. No symptoms, arrhythmias, or ECG changes occurred. Colchicine was discontinued 4 weeks after the second dose. The clinical course was uneventful. SARS-CoV-2-S-IgG/M antibody levels 5 weeks after second vaccination were >2,500 U/mL. Subsequently, the patient could successfully complete the vaccination course with a third dose of mRNA-1273, again under colchicine prophylaxis for 4 weeks and without evidence of myopericarditis. Figure 2 shows the timeline of the clinical milestones of the patient.

## Discussion and conclusion

We present a case of myopericarditis in temporal association with a first dose of an mRNA COVID vaccine. The patient had classical pericarditis and a substantial dynamic troponin elevation. The troponin level was elevated before an uneventful diagnostic angiography without intervention was performed. The cardiac MRI 2 days following the angiography showed no evidence of ischemia or alternative causes of the troponin elevation. Based on the case definitions for vaccine-associated myocarditis by the Brighton Collaboration, the case fulfilled the criteria for “probable myocarditis” in addition to pericarditis (3).

Pericarditis following mRNA COVID vaccinations has been reported, although actual incidence rates have not yet been established (4, 5), likely due to a mix between isolated myocarditis and myopericarditis (5). In a case series, Ochs et al. reported five cases of isolated pericarditis occurring within 7 days following COVID vaccination (6). In contrast to our case, these subjects had normal, or borderline elevated, troponin levels. Vaccine-induced myocarditis emerged as a rare but serious adverse event following immunization with mRNA—vaccines against SARS-CoV-2 during the postmarketing surveillance (7, 8). While no cases occurred in the pivotal phase III studies trials (8, 9), epidemiological data estimated the incidence of SARS-CoV-2 vaccine-associated myocarditis between 0.3 and 5.5 per 100 per 100,000 vaccinated persons (7, 10–16). Data from different studies are summarized in Table 2. Most frequently, mRNA vaccine-induced myocarditis occurred in young male (<40 years of age) 1–5 days after the second dose of an mRNA vaccine with higher incidence following mRNA-1273 than BNT162b. The clinical course was mostly mild with only a few patients requiring steroids or intensive care treatment while the majority responded well to NSAIDs (14). So far, no further risk factors for the onset of vaccine-induced myocarditis could be identified.

**Table 2:** Reported incidence of myocarditis among general population after at least one dose of after SARS-CoV-2 vaccine.

Incidence per 100,000 people	Study population, *n* (median age)	Country	Period	Vaccines	Type of study	References
1.90	38,615,491 (≥12 years)	United Kingdom	December 1, 2020 to August 24, 2021	Comirnaty, Spikevax, Vaxzevria	Self-controlled case series study	Patone, *Nature* 2021 (7)
2.13	2,558,421 (≥16 years)	Israel	December 20, 2020 to May 24, 2021	Comirnaty	Retrospective cohort study	Witberg, *NEJM* 2021 (10)
5.54	5,442,696 (≥16 years)	Israel	December 20, 2020 to May 31, 2021	Comirnaty	Retrospective cohort study	Mevorach, *NEJM* 2021 (11)
1.38	4,931,775 (≥12 years)	Denmark	October 1, 2020 to October 5, 2021	Comirnaty, Spikevax	Population based cohort study	Husby, *BMJ* 2021 (12)
0.29	10,162,227 (≥12 years)	United States of America	December 14, 2020 to June 26, 2021	Comirnaty, Spikevax	Interim analyses of surveillance monitoring of mRNA COVID-19 vaccines	Klein, *JAMA* 2021 (13)
0.85	192,405,448 (≥12 years)	United States of America	December 14, 2020 to June 26, 2021	Comirnaty, Spikevax	Descriptive study of reports of myocarditis to the Vaccine Adverse Event Reporting System (VAERS), national passive reporting system	Oster, *JAMA* 2022 (14)

SARS-CoV-2 vaccine: Comirnaty (BNT162b2, Pfizer/BionTech), Spikevax (mRNA-1273, Moderna), or Vaxzevria (ChAdOx1-S, AstraZeneca).

To the best of our knowledge, this is the first case report on mRNA vaccine-induced myopericarditis in a patient who underwent allogeneic HCT. This case shows various specific peculiarities. Our patient was already 70 years old, and the symptoms developed about 12 h after vaccination. Although young men seem to be at the highest risk and the median time to symptom onset is 2–3 days, in real life, the age range of persons affected by mRNA vaccine-associated myocarditis is much broader, and a considerable number of individuals become symptomatic within the first 24 h (14, 17).

The pathogenesis of myopericarditis associated with mRNA vaccination is not entirely understood but may involve a dysregulation of several immunological pathways, including mRNA-induced innate immune stimulation *via* Toll-like receptors, hypersensitivity reaction against vaccine components, molecular mimicry between cardiac self-antigen and the spike protein of SARS-CoV-2, or hormone-dependent alteration of inflammatory pathways (testosterone vs. estrogen) (7, 18–20). Recently, autoantibodies against the interleukin-1 receptor antagonist (IL-1RA) were found in histologically proven myocarditis cases after SARS-CoV-2 vaccination (21). Given the early onset of symptoms after a single dose of the mRNA and in the absence of a previous SARS-CoV-2 infection, it is unlikely that vaccine-induced adaptive immune mechanisms were involved in our case. Previously, an in-depth immunological analysis of a case of mRNA vaccine-associated myocarditis revealed a dysregulation of the innate immune system underlying the pathogenesis of myocarditis (19). The clinical response of our patient to colchicine, an inhibitor of the innate immunity, is well matching this hypothesis. Notably, the transplanted immune system could have interfered with those mechanisms contributing to this side effect presentation. Because this patient underwent allogeneic HCT, we also considered a flare of GvHD in differential diagnosis. Pericarditis as a sign of chronic GvHD is rare, usually manifests as part of a polyserositis and/or other signs of GvHD. Because systemic immunosuppression has already been stopped 7 months prior to the event of myopericarditis and no other clinical signs of GvHD occurred, we consider a GvHD flare highly unlikely.

**Figure 3:**
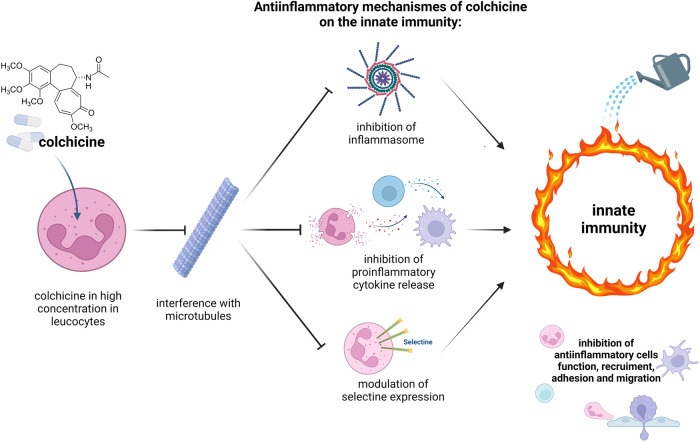
Mechanisms of action of colchicine on the innate immunity. High cytoplasmic concentration of colchicine especially in neutrophils are reached because of physiological reduced expression of transport P-glycoprotein (P-gp), which usually excretes drugs out of the cells. Colchicine inhibits assembly and attachment of microtubules. Consequently, several mechanisms of anti-inflammatory cell of the innate immunity are inhibited: inflammasome, a cytosolic multiprotein oligomers complex, which should activate an inflammatory cascade; release of proinflammatory cytokines (such as IL-1-β and IL-18) are downregulated; the expression E- and L-selectine on neutrophil surface, which promote the adhesion and the migration of those cells, are inhibited. This complex and sophisticated model results into inhibition of anti-inflammatory cells functions, recruitment, and motility of the innate immunity (22, 23, 25) (figure created with biorender.com).

Our patient presented with a severe clinical course requiring longer in-hospital management because of prolonged precordial pain and delayed troponin normalization. Colchicine is a standard of care for patients with pericarditis (22–25). While less frequently used for the treatment of myopericarditis, a recent study indicated better outcome of myopericarditis if treated with colchicine (26). Based on (i) the observation that the inflammasome pathway, that is targeted by colchicine (Figure 3), is involved in the pathogenesis of mRNA vaccine-associated myocarditis (1), and (ii) the reported reduced risk of gout flares in association with mRNA vaccinations in patients treated with colchicine (2), we speculated that colchicine may reduce the risk for symptom reoccurrence upon re-exposure to an mRNA vaccine. Indeed, colchicine seemed to be effective for both acute treatment and prophylaxis, allowing a safe re-exposure to the mRNA vaccine and thereby the completion of the immunization resulting in an adequate SARS-CoV-2-S-antibody response. Whether colchicine reduces the risk for mRNA vaccine-associated pericarditis and myocarditis should be assessed in larger patient cohorts.

Data on vaccine-re-exposure in patients experiencing a myopericarditis after a previous single dose of vaccination are scare (27). Due to the high individual risk for severe COVID and the ongoing pandemic situation, we decided to re-expose the patient to a second dose with the same vaccine (mRNA-1273 Spikevax; Moderna) but under colchicine prophylaxis. At that time only, mRNA vaccines (mRNA-1273 and BNT162b) were available in Switzerland; therefore, no switching to another class of vaccine was possible. At the time of the second vaccination, no data were available about the differences in the rate of myopericarditis among mRNA vaccines.

This report has some limitations: first, it is a single-case report of an immunocompromised patient after allogeneic HCT, thus precluding generally valid conclusions of our observations. Second, the presentation differed from published cases of mRNA vaccine-induced myocarditis by the absence of typical myocarditis findings, such as late gadolinium enhancement and edema, in the cardiac MRI. However, given the high troponin indicating myocardial injury and the extensive work-up to exclude alternative causes, probable myopericarditis could be diagnosed, according to the proposed case definition of the Brighton Collaboration (3). In line with this, the patient had chest pain over several days, and the troponin levels showed an increase to a peak 5 days following vaccination. Third, we cannot exclude that second dose was well-tolerated independent of colchicine prophylaxis. Finally, we only assessed humoral immunogenicity after the second dose and therefore cannot comment on whether colchicine may have affected the adaptive T-cell response to the vaccination in this patient. However, colchicine is generally not considered to interfere with adaptive immunity (Figure 3).

Our observation suggests that, following careful individual risk–benefit assessment, re-exposition can be safe under prophylaxis with colchicine inducing sufficient immunization thus reinforcing our conviction to continue a uniform and widespread vaccination campaign to protect our fragile patient population.

## Data Availability

The original contributions presented in the study are included in the article/**Supplementary Material**. Further inquiries can be directed to the corresponding author.

## Supplementary material

The Supplementary Material for this article can be found online https://www.frontiersin.org/articles/10.3389/fcvm.2023.1135848/full#supplementary-material.
