# Supplementary figures and images for: Case report: mRNA-1273 COVID-19 vaccine-associated myopericarditis: Successful treatment and re-exposure with colchicine
Source: Front Cardiovasc Med. 2023 Apr 17;10:1135848. doi: 10.3389/fcvm.2023.1135848 (PMC10149711; doi:10.3389/fcvm.2023.1135848)

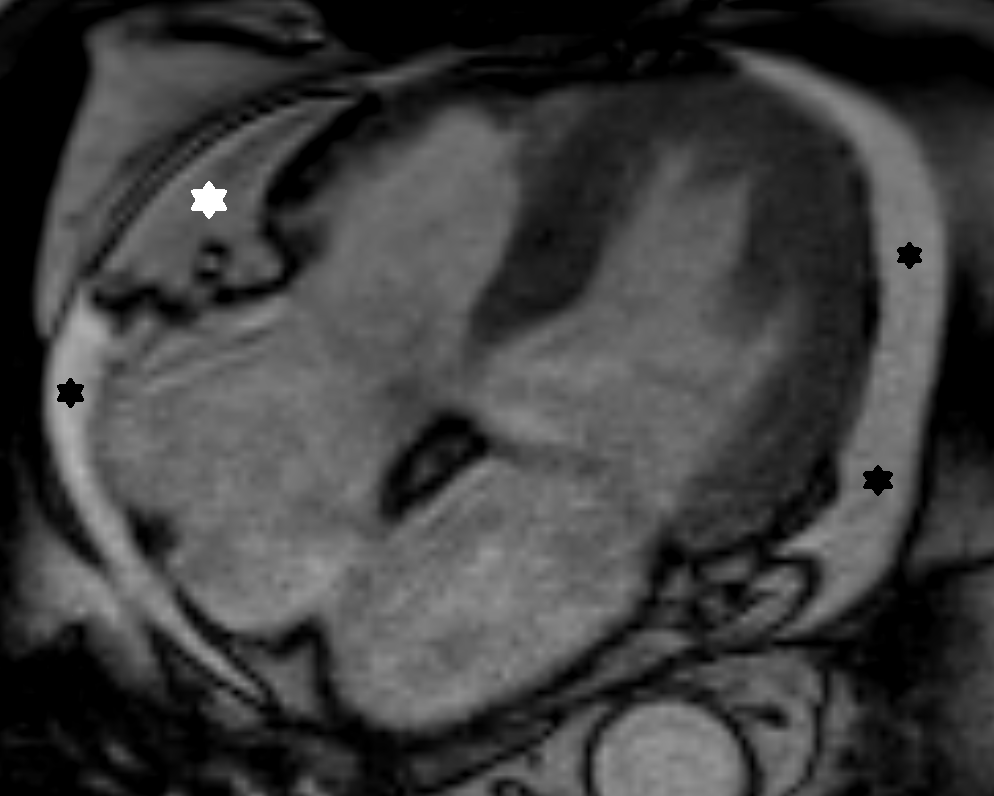

Supplement: Supplementary file 2 [file Image1.png]

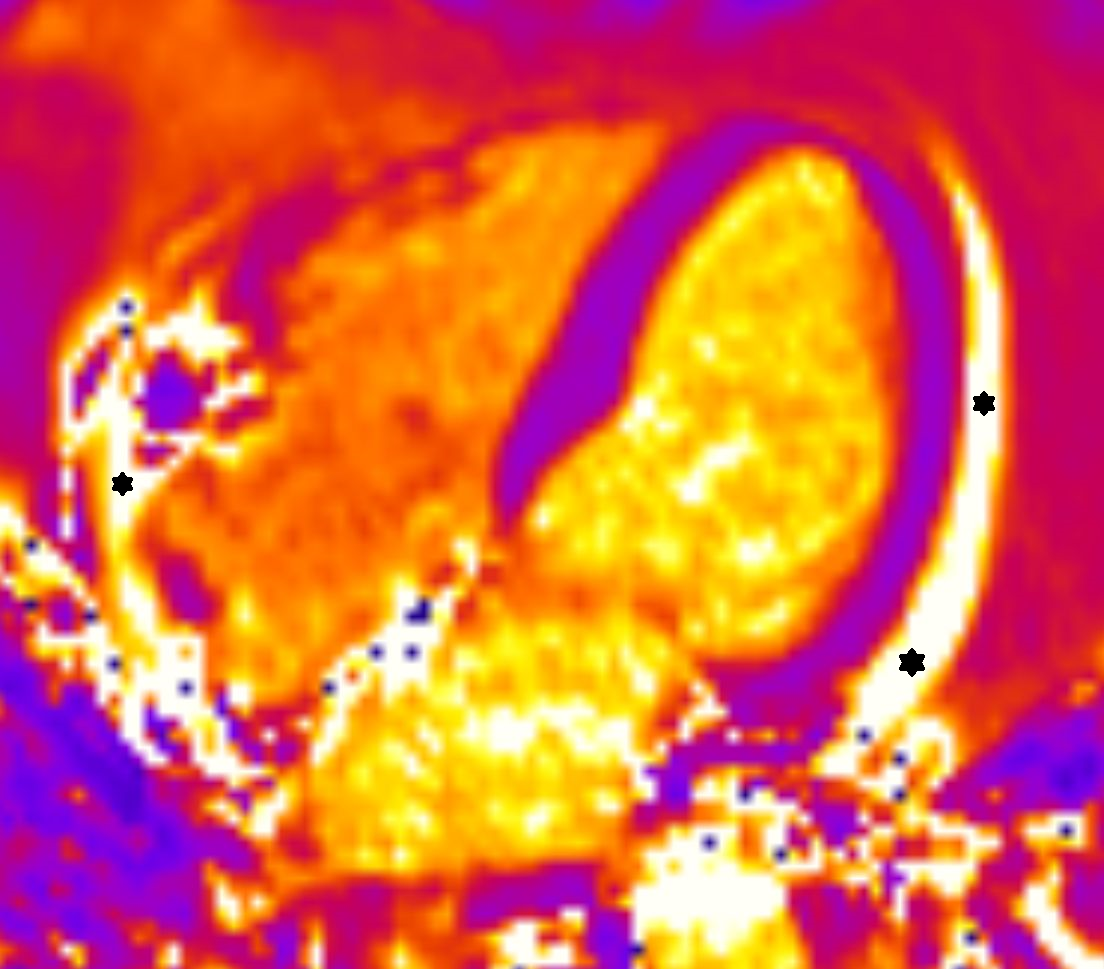

Supplement: Supplementary file 3 [file Image2.png]

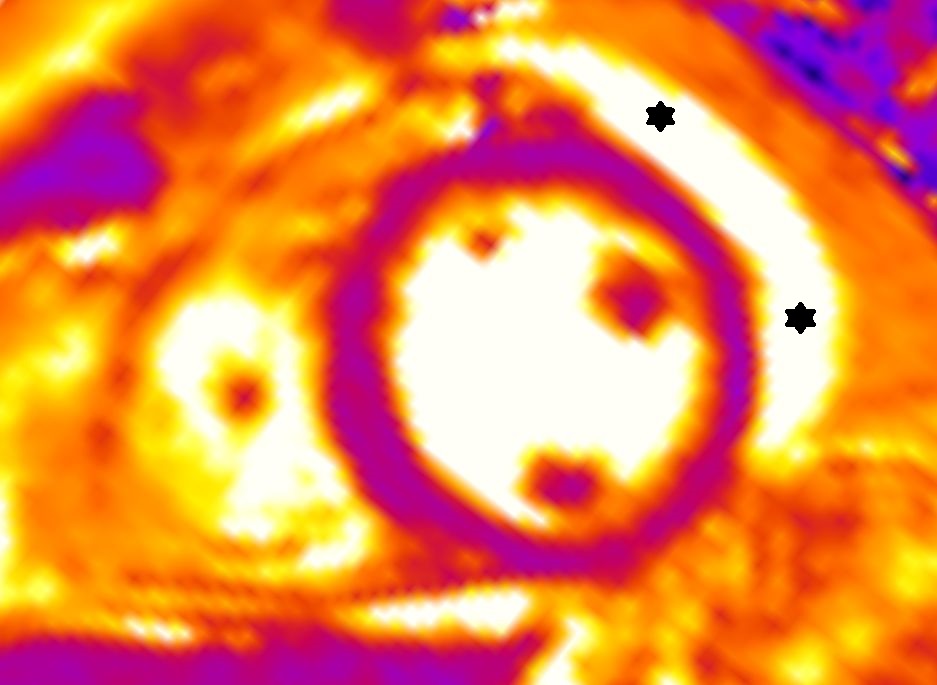

Supplement: Supplementary file 4 [file Image3.jpeg]
